# Molecular Portrait of GISTs Associated With Clinicopathological Features: A Retrospective Study With Molecular Analysis by a Custom 9-Gene Targeted Next-Generation Sequencing Panel

**DOI:** 10.3389/fgene.2022.864499

**Published:** 2022-04-25

**Authors:** Haoran Qian, Na Yan, Xiaotong Hu, Junchang Jiang, Zhengzheng Cao, Dan Shen

**Affiliations:** ^1^ Department of General Surgery, Sir Run Run Shaw Hospital, School of Medicine, Zhejiang University, Hangzhou, China; ^2^ Dian Diagnostics Group Co., Ltd., Hangzhou, China; ^3^ Key Laboratory of Clinical in Vitro Diagnostic Techniques of Zhejiang Province, Hangzhou, China; ^4^ Department of Pathology, Sir Run Run Shaw Hospital, School of Medicine, Zhejiang University, Hangzhou, China

**Keywords:** gastrointestinal stromal tumors, molecular subtypes, clinicopathological features, next-generation sequencing, target therapy

## Abstract

**Objectives:** The study aims to investigate genetic characterization of molecular targets and clinicopathological features with gastrointestinal stromal tumors based on targeted next-generation sequencing.

**Materials and Methods:** We selected 106 patients with GISTs from Sir Run Run Shaw Hospital between July 2019 and March 2021. FFPE samples and paired blood samples were obtained from these patients who underwent excision of the tumor. A customized targeted-NGS panel of nine GIST-associated genes was designed to detect variants in the coding regions and the splicing sites of these genes.

**Results:** In total, 106 patients with a GIST were included in the study which presented with various molecular driver alterations in this study. *KIT* mutations occurred most often in GISTs (94/106, 95.92%), followed by point mutations in *PDGFRA*. *KIT* or *PDGFRA* mutations were detected to be mutually exclusive in the GIST. A total of eight patients with wide-type *KIT/PDGFRA* were characterized as WT-GISTs, according to clinical diagnosis which included six quadruple-WT GISTs, 1 *BRAF*-mutant, and 1 *NF1*-mutant GIST. In *KIT* exon 11, the most common mutation type was the codon Mutation (in-frame deletion or indels), whereas the missense mutation was the dominant type in *KIT* exon 13 and *KIT* exon 17. All variations in *KIT* exon 11 observed in this study were concentrated at a certain position of codon 550 to codon 576. Mutation in *KIT* exon 9 was mostly located at codon 502–503. Two germline pathogenic mutations were detected: NF1-R681* and KRAS-T58I. NF1-L591P was a germline mutation to be identified for the first time and is not recorded in the database. The frequency of driving mutations differed between the primary anatomical site in the GIST (*p* = 0.0206). *KIT* exon 11 mutants had a lower proliferation index of Ki67 (68.66%,≤5%), while 50.00% of *KIT* exon 9 mutants had the Ki67 status greater than 10%.

**Conclusion:** The occurrence and development of a GIST is driven by different molecular variations. Resistance to TKIs arises mainly with resistance mutations in *KIT* or *PDGFRA* when they are the primary drivers. Targeted NGS can simultaneously and efficiently detect nine GIST-related gene mutations and provide reference for clinicians’ individualized diagnosis and treatment. Our results have important implications for clinical management.

## Introduction

A gastrointestinal stromal tumor (GIST) is the most common mesenchymal malignancy of the gastrointestinal tract which originates from Cajal cells of the digestive tract and account for 3% of gastrointestinal malignant tumors. The most common clinical manifestation of a GIST is a gastric tumor or a bowel tumor. The rectum, colon, esophagus, and other sites are rare (Ducimetière et al., 2011; Boonstra et al., 2018; Florindez and Trent, 2020; Virgilio et al., 2021). Pathological examination is the most reliable method for the diagnosis of a GIST (Blay et al., 2021a). National Comprehensive Cancer Network (NCCN) guidelines recommend endoscopic ultrasound with fine-needle aspiration and biopsy (EUS-FNAB) as the first choice (Zhao et al., 2020). The expression of tumor markers in tumor tissues was detected by immunohistochemistry (Fernández et al., 2018). c-Kit (stem cell growth factor receptor, CD117) is a protein encoded by the *KIT* gene in humans. CD117 is the most important IHC marker which is expressed in 85–95% of GISTs (Ramdani et al., 2020). DOG1 is a useful marker for these tumors that the GIST does not express *KIT* on IHC. Other tumor markers include CD34, smooth muscle action (SMA), etc., (Luo et al., 2004; Malik et al., 2019; Bradea et al., 2021).

In total, 60–70% GISTs can acquire mutation of *KIT*, and 10–15% GISTs acquire mutation of platelet-derived growth factor receptor A (PDGFRA) that both promote to the occurrence and development of GISTs (Joensuu et al., 2013; Joensuu et al., 2015; Nishida et al., 2016). *KIT* and *PDGFRA* mutations play a crucial role in the pathogenesis of GISTs (Demetri et al., 2006). Chinese consensus guidelines for diagnosis and management of gastrointestinal stromal tumor recommend *KIT/PDGFRA* gene testing for CD117/DOG1-negative GIST patients, which acts as a supplement for immunohistochemical diagnosis (Li et al., 2017).

GIST is divided into three types at the molecular level based on the mutations of *KIT* and *PDGFRA*: GIST with *KIT* mutations, GIST with *PDGFRA* mutations, and non*-KIT or PDGFRA* somatic mutation (WT-GIST) (Daniels et al., 2011). WT-GIST is complex due to the existence of different subgroups with distinct molecular hallmarks. About 30% of WT-GISTs show deletion mutations of succinate dehydrogenase subunit A (SDHA) (Boikos et al., 2016). Other molecular hallmarks include mutations of neurofibromatosis type 1 (NF1), *BRAF*, or *RAS* (Corless et al., 2011a; Blay et al., 2021a). It follows that GIST is a cancer with comparatively small genetic heterogeneity. The cancer-driven pathway of a GIST is a downstream signaling pathway mediated by KIT/PDGFRA receptors (Blay et al., 2021b). The precise treatment of the cancer gene map for GISTs has become increasingly mature.

The tyrosine kinase inhibitor (TKI) imatinib is a model of targeted therapy for GISTs which can be used to treat GISTs with *KIT/PDGFRA* mutation (Park et al., 2014; Gang and Wang, 2018). However, the therapeutic response and dosage of GIST to tyrosine kinase inhibitors are closely bound up with molecular subtypes (Blay et al., 2021b). Specifically, the *KIT* exon 11 mutation is more responsive to imatinib treatment than the *KIT* exon 9 mutation or WT-GISTs. The *KIT* exon 9 mutation requires double dose of imatinib (800 mg/d) (Nishida et al., 2015; Reichardt, 2016). KIT-V654A and KIT-T670I mutations are resistant to imatinib (Guo et al., 2007). PDGFRA-D842V mutations are also characterized broadly as imatinib resistance mutations which can adjust the drug treatment strategy to 300 mg/d avapritinib (Jun et al., 2018; Heinrich et al., 2020; Smrke et al., 2020; Jones et al., 2021). Therefore, it is necessary to understand the molecular characteristics before tyrosine kinase inhibitor treatment to ensure the optimal treatment strategy. The purpose of this study was to investigate the relationship between the molecular variation and clinicopathological features in GIST patients by targeted next-generation sequencing (NGS), in order to deepen the understanding of GIST-individualized treatment.

## Materials and Methods

### Patients and Tumor Samples

A total of 106 solid tumor samples from gastrointestinal stroma and paired blood samples were analyzed by a custom 9-gene targeted next-generation sequencing panel, which was obtained from Run Run Shaw Hospital between July 2019 and May 2021. All specimens were pathologically and immunohistochemically confirmed as GISTs. Tumor cells accounted for more than 20% of the tumor population. Clinical data of all patients were collected and sorted out on the basis of age, gender, tumor location, tumor size, mitotic count, immunohistochemical detection index (CD117, CD34, DOG1, S-100, Ki-67, SDHB, SMA, and desmin), etc., (Supplementary Table S1). This study was approved by the internal review board of the Run Run Shaw Hospital.

### Sample Preparation and DNA Extraction

The pathologist performed a histological assessment with hematoxylin and eosin-stained sections to confirm the tumor purity. Then, the tumor areas of the FFPE sections were macrodissected. Tumor cells accounted for more than 20% of the tumor population. Genomic DNA from FFPE samples of the GIST were extracted by using a QIAamp DNA FFPE Tissue Kit (QIAGEN, Dusseldorf, Germany), following the manufacturer’s instructions. Paired blood samples of GIST were extracted using a QIAamp DNA Blood Midi Kit (QIAGEN, Dusseldorf, Germany), according to manufacturer’s instructions. The DNA concentration was measured using a Qubit 3.0 (Thermo Fisher Scientific, Waltham, United States) fluorometer. The size distribution of DNA was analyzed using a Qsep100 (Bioptic, Taiwan, China) system.

### Next-Generation Sequencing Library Preparation

This study used the CleanPlex™ (Paragon Genomics, Silicon Valley, United States) panel with an optimized manufacturer’s protocol to prepare sequencing libraries. 40 ng of genomic DNA was enriched in the target region of nine GIST-related genes by multiplex PCR and then ligated with indexed sequencing adapters sequentially. Purification of DNA libraries used Agencourt AMPure XP beads (Beckman Coulter, United States). The purified NGS library was quantified using the Qubit 1×dsDNA Assay Kit (Thermo Fisher Scientific, Waltham, United States), and its fragment size distribution was analyzed using a Qsep100 (Bioptic, Taiwan, China) system. A nine GIST-related gene panel was used to identify one or more single-nucleotide variants, insertions, deletions, duplications, and delin mutations. The nine genes include *KIT*, *PDFRA*, *KRAS*, *BRAF*, *NF1*, *SDHA*, *SDHB*, *SDHC*, and *SDHD*.

### Sequencing and Bioinformatics Analysis

In order to ensure the reliability and validity of the experimental results, all library construction and sequencing were completed in a CAP-certified laboratory. Sequencing was performed on the Illumina NextSeq 500 platform (Illumina, San Diego, United States). The mean coverage depth is approximately >1000X for the tumor samples and >30X for the paired blood samples. A minimal variant frequency of 5% was designated as a mutation. The paired-end sequencing data of the libraries in the FASTQ format were mapped to the human genome (hg19) by the Burrows–Wheeler Aligner (BWA-MEM). MuTect19 with default parameters was applied to paired blood and tumor BAM files for identification of somatic single-nucleotide variants (SNV). Small insertions and deletions (indels) were detected by SCALPEL. SNV and indel annotation was performed by ANNOVAR21 using the hg19 reference genome and 2014 versions of standard databases and functional prediction programs.

### Statistical Analysis

The statistical package stats of R version 4.1.1 software were used for statistical analysis. Continuous variables were reported as mean and standard deviation or median and interquartile range and compared by using the Student t test or Mann–Whitney U test. Chi-square analysis was performed toward analyses of subgroups, and Fisher’s exact test was used in cases of small numbers. All tests were 2-sided, with *p* ≤ 0.05 as the criterion standard for determining significance. Structural changes induced by amino acid substitution were predicted by Missense3D software. A clustering correlation heatmap with signs was performed using the Kendall correlation analysis. GO enrichment analysis and KEGG pathway analysis were established with OmicShare tools.

## Results

### Clinicopathological Characteristics of Patients

The total of 106 patients were included in this study, of which 73 were primary tumors (68.87%) and 33 were recurrent disease (31.13%) (Table 1). Gender disparity in GIST incidence was not observed as the male-to-female ratio is 1.3:1 (60 male and 46 female patients). The age of the first operation composition ranged from 35 to 89 with a median age of 58. GIST arose in the non-stomach (64, 60.38%) sites more than the stomach sites (42, 39.62%). Location of the disease disparity in GIST incidence was not observed as the stomach-to-non-stomach ratio is 1:1.03. The level of the mitotic phase in the stomach is higher (Mitotic count [x/HPF] > 5, 32.43%), which is different from that of non-gastric primary GIST (Mitotic count [x/HPF] > 5, 8.33%;*p*-value = 0.0389). There was no significant difference between the location of the primary focus and tumor size (Supplementary Figure S1, Supplementary Table S1).

**TABLE 1:** Demographic and clinical characteristics of 106 patients with GIST.

Characteristics	No. of patients	%	Characteristic	No. of patients	%
**Gender**			**CD34**		
Male	60	56.60	positive	84	79.25
Female	46	43.40	negative	12	11.32
			unknown	10	9.43
**Mean age of first operation [years] (range)**		58 (35–89)	**SMA**		
≤55	37	34.91	positive	9	8.49
>55	69	65.09	negative	86	81.13
			unknown	11	10.38
**Location of primary GIST**			**S-100**		
Stomach	42	39.62	positive	1	0.94
Others	64	60.38	negative	95	89.62
			unknown	10	9.43
**Tumor size (cm)**			**Desmin**		
≤2	6	5.66	positive	2	1.89
>2,<5	41	38.68	negative	93	87.74
≥5	58	54.72	unknown	11	10.38
unknown	1	0.94			
**Mitotic count [x/HPF]**			**SDHB**		
≤5/HPF	57	53.77	positive	90	84.91
>5/HPF	29	27.36	negative	4	3.77
unknown	20	18.87	unknown	12	11.32
**CD117**			**Proliferation index of Ki67 (%)**		
positive	94	88.68	≤5	60	56.60
negative	3	2.83	>5,< 10	11	10.38
unknown	9	8.49	≥10	25	23.58
**DOG1**					
positive	85	80.19	**Disease status**		
negative	12	11.32	primary tumors	73	68.87
unknown	9	8.49	recurrent disease	33	31.13

### Gene Mutation Distributions and Frequencies

**FIGURE 1:**
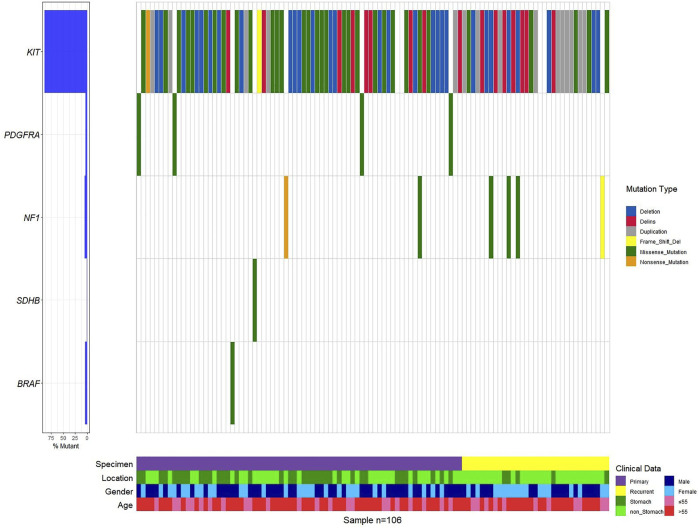
Genetic profile of the GIST.

Mutations in 106 patients with primary and recurrent GISTs have a preference (Figure 1, Supplementary Figure S2, Supplementary Table S1). *KIT* mutations occurred most often in GISTs (94 of 106 tumors, 95.92%), followed by the point mutation in *PDGFRA.* This predominant preference presented both in primary and recurrent GISTs (Supplementary Table S1, Figure 1, Supplementary Figure S2). *KIT* mutations or *PDGFRA* mutations were detected to be mutually exclusive here, which is in consistency with previous reports (von Mehren and Joensuu, 2018a; Blay et al., 2021a). A total of eight patients with wide-type *KIT/PDGFRA* were characterized as WT-GISTs, according to clinical diagnosis. These include six quadruple-WT (*KIT/PDGFRA/SDH/RAS*-WT) GISTs, 1 *BRAF-*mutant, and 1 *NF1*-mutant GIST.

**FIGURE 2:**
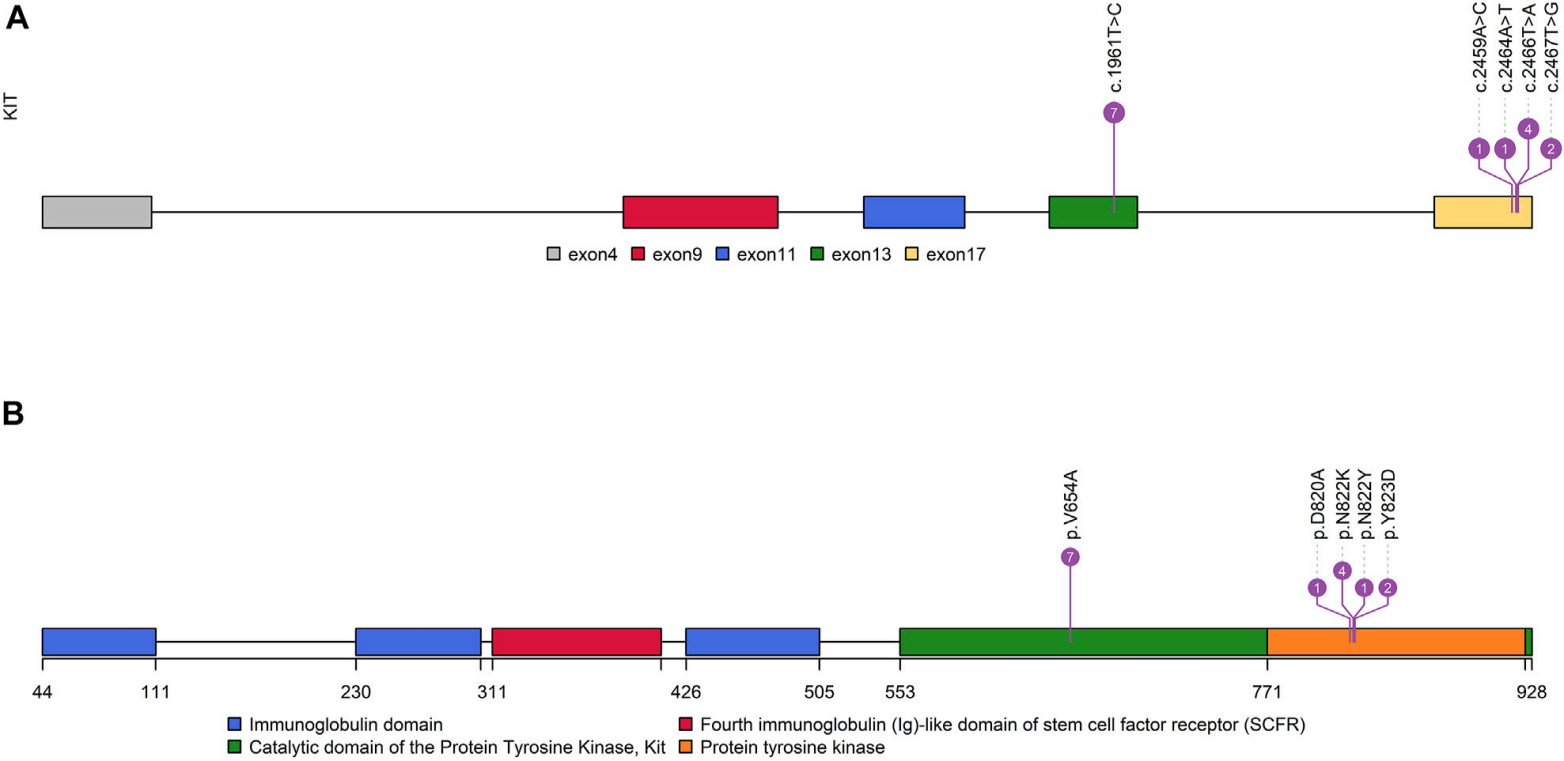
Distribution characteristics of *KIT*-resistant mutations. **(A)**: Distribution of resistant mutations in the *KIT* gene. **(B)**: Distribution of resistant mutations in KIT functional domains.

It is worth noting that none of the first-episode patients developed resistance mutations, except for one patient who had been treated with IM 400 mg neoadjuvant therapy for 25 months preoperatively. This patient only detected *KIT* exon 11 p.W557_K558 deletion before IM therapy (Supplementary Table S1). However, we found secondary resistance mutations (*KIT* exon 13 p.V654A, exon 17 p.D820V, p.N822K/Y, and p.Y823D) in 13 recurrent patients (Figure 2, Supplementary Table S1, Supplementary Figure S2). A total of 14 patients with resistant mutations were detected in this study, and 92.86% of them relapsed.

### KIT-Mutant GISTs

*KIT* mutations including deletion, deletion-insertion (indels), duplication, and missense mutations occurred mostly in exon 11 (74 of 94 *KIT*-mutant GISTs), followed by exon 9, exon 13, exon 17, and exon 4 (Supplementary Figure S2, S3A). In exon 11, the most common mutation type was the codon mutation (in-frame deletion or indels), whereas the missense mutation was the dominant type in exon 13 and exon 17 (Supplementary Figure S2). This preference of variant type in different exons of *KIT* was statistically significant (*p* = 6.011e-07) (Table 2).

**TABLE 2:** The genomic features of 106 GIST patients.

Features		Variation type					
			Point mutation		Codon mutation		
		Number of patients %	N	%	N	%	*p* Value
Wild-type	8	7.55%	3	37.50%	1	12.50%	0.4900
Mutation	98	92.45%	49	50.00%	61	62.24%	
KIT	94	95.92%	44	46.81%	61	64.89%	6.011e-07
exon 4	1	1.06%	0	/	1	1.06%	
exon 9	15	15.96%	1	6.67%	14	14.89%	
exon 11	74	78.72%	28	37.84%	48	51.06%	
exon 13	9	9.57%	9	100.00%	0	/	
exon 17	7	7.45%	7	100.00%	0	/	
PDGFRA	4	4.08%	4	100.00%	0	/	
exon 18	4	100.00%	4	100.00%	0	/	

The deletions of codon 557–558 (c.1669-1674del) in exon 11 of *KIT* were 63.04% in 46 patients, which is associated with the malignant behavior as mentioned in reports (Martin-Broto et al., 2010; Joensuu et al., 2015). The next most frequent mutation in exon 11 of *KIT* was the missense mutation of codon 559 (c.1676T > A/C/G). All variations in *KIT* exon 11 observed in this study were concentrated at a certain position of codon 550 to codon 576 (Supplementary Table S1), which may be defined as the hotspot region of GISTs.

**FIGURE 3:**
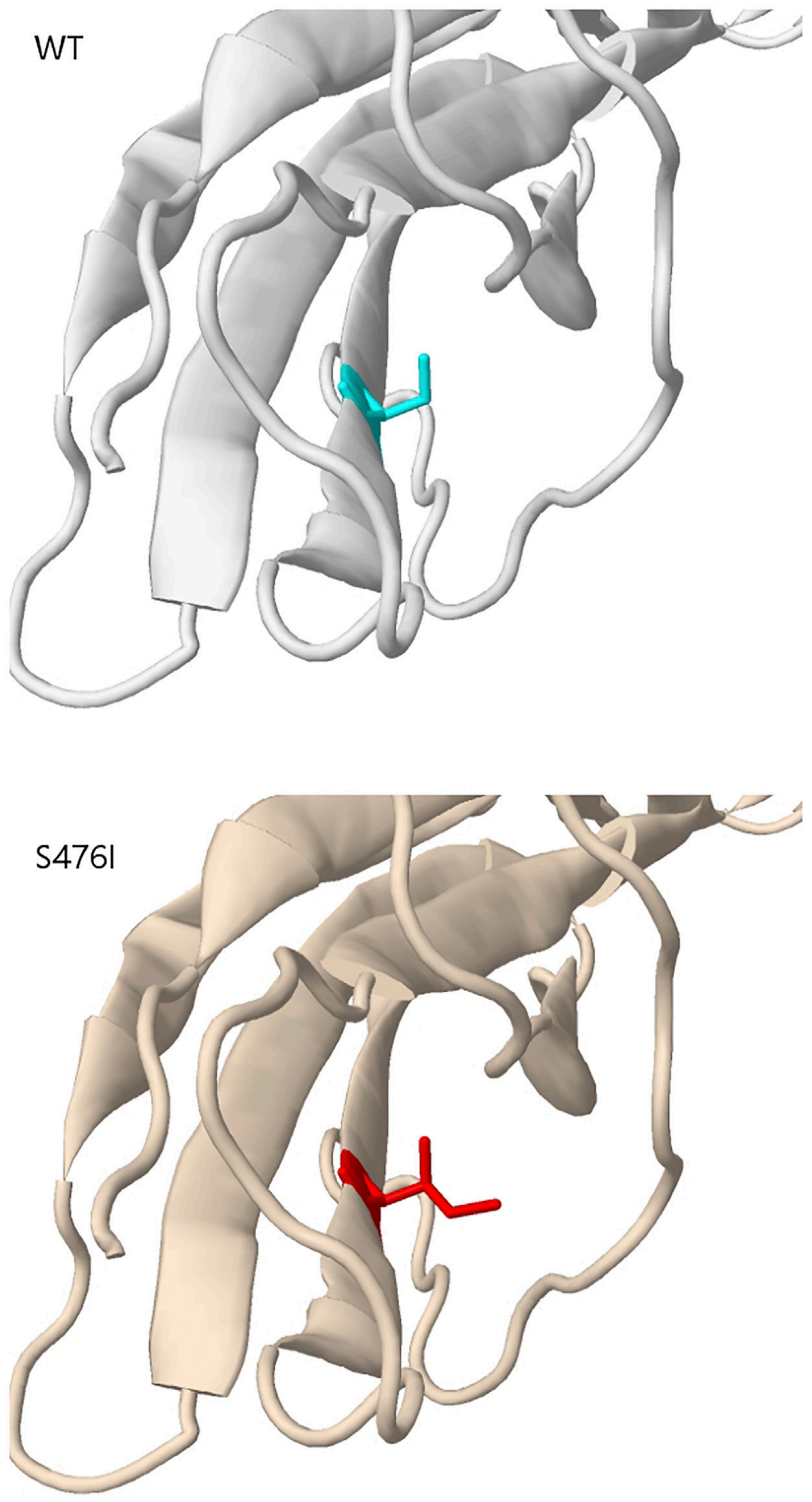
Missense3D predicts the tertiary structure changes introduced by KIT p.Ser476Ile.

Mutation in *KIT* exon 9 mostly located at codon 502–503. The duplication insertion of A502-Y503 codons account for 93.33% (14 of 15 GIST) GISTs, which was identified a variation in *KIT* exon 9. Besides, a variant at codon 476 (c.1427G > T, p.Ser476Ile) of unknown significance in *KIT* exon 9 was found in this study. Structural changes induced by amino acid substitution were predicted by Missense3D software. The results showed that the variation of Ser to Ile at position 476 resulted in hydrogen bond damage and atomic collision with surrounding amino acid residues. It was inferred that KIT-S476I may be a pathogenic mutation (Figure 3). Among 15 GIST patients who harbored the *KIT* exon 9 mutation, nine (60%) were recurrence patients, indicating a higher risk of relapse after surgical excision of exon 9-mutated GIST patients.

The missense mutation affecting codon V654 (c.1961T > C) in exon 13 of *KIT* was identified only in recurrence GISTs in this study. This finding is consistent with the previous understanding that V654A in exon 13 is the secondary resistance mutation acquired under the therapeutic pressure of a TKI (Nishida et al., 2008; Serrano et al., 2019). Codon L642 (c.1924A > G) in exon 13 presented in two primary GIST patients in this study.

In exon 17 of *KIT*, D820A, N822K/Y, and Y823D were observed in seven recurrent patients and one primary patient (Figure 2). As the secondary mutation, the activation loop mutation in exon 17 (e.g., D820A) stabilizes the active formation of KIT kinase, thus contributing resistance of TKI. The mutation observed in exon 17 in our study was also considered as the cause for relapse.

### PDGFRA-Mutant GISTs

PDGFRA-D842V was observed in all (4/4) of GISTs with *PDGFRA* alterations in this study (Supplementary Table S1). It means that these patients cannot benefit from imatinib treatment.

### Germline Mutation in GISTs

Two germline pathogenic mutations were detected, NF1-R681* and KRAS-T58I. We also obtained eight germline mutations of unknown clinical significance, SDHA-Y211H, SDHC-V9I, SDHB-A6S, BRAF-G69A, PDGFRA-S716R/D756N, and NF1-R2713Q/Y1292C. NF1-L591P was a germline mutation to be identified for the first time and is not recorded in the database (Supplementary Table S2).

### Associations Between the Molecular Subgroup and Clinicopathological Characteristics of GISTs

The frequency of driving mutations differed between primary anatomical sites in GISTs (*p* = 0.0321) (Table 3). GISTs bearing *KIT* exon11 mutations were located most often in the gastrointestinal tract (53.6%, 30/56) and small intestine (35.7%, 20/56). Mutations in exon 9 of *KIT-*mutant GISTs all happened in the small intestine (100%, 6/6) in our cohort. In the primary rectal GIST, the most frequent driver mutations occurred in *KIT* exon 11 (100%, 5/5). All GISTs harboring the PDGFRA-D842V mutation had a unique gastric primary localization. The wide-type *KIT/PDGFRA* GISTs arose in the small intestine and stomach.

**TABLE 3:** Clinicopathological feature correlation with molecular classification.

Characteristics	KIT	PDGFRA	Wild-type	*p* Value
**Gender**				
Male	53	3	4	0.7065
Female	41	1	4	
**Age**				
≤55	33	2	2	0.6879
>55	61	2	6	
**Location of GIST**				
Stomach	36	4	2	0.0321
Others	58	0	6	
**Tumor size (cm)**				
≤2	4	1	1	0.2187
>2,<5	36	1	4	
≥5	53	2	3	
**Mitotic count [x/HPF]**				
≤5/HPF	50	3	1	0.0563
>5/HPF	28	0	4	
**CD117**				
positive	86	3	5	0.0274
negative	1	0	2	
**DOG1**				
positive	77	3	5	0.4762
negative	10	0	2	
**CD34**				
positive	77	1	6	0.03637
negative	9	2	1	
**SMA**				
positive	9	0	0	1
negative	76	3	7	
**S-100**				
positive	1	0	0	1
negative	85	3	7	
**Desmin**				
positive	2	0	0	1
negative	83	3	7	
**SDHB**				
positive	81	3	6	0.3673
negative	3	0	1	
**Proliferation index of Ki67 (%)**				
≤5	52	3	5	0.7805
>5,< 10	10	0	1	
≥10	24	0	1	

There was a significant difference in the *KIT* mutation rate among GIST patients in the Ki67 status group of ≤5, group of >5 and <10, and group of ≥10 (*p* = 0.001333) (Table 4). *KIT* exon11 mutants had a lower proliferation index of Ki67 (68.66%,≤5%), while 50.00% of *KIT* exon 9 mutants had a Ki67 status greater than 10%. Mutations in KIT 9 exon were more likely to occur in non-gastric areas (Table 4).

**TABLE 4:** Features of KIT exons.

Features	Number of patients	%	Location of GIST		Mitotic count [x/HPF]		Tumor size (cm)			Proliferation index of Ki67 (%)		
			Stomach	Non-stomach	≤5/HPF	>5/HPF	≤2	>2,<5	≥5	≤5	>5,< 10	≥10
KIT	94								p-value = 0.000209	p-value = 0.1069	p-value = 0.3118	p-value = 0.001333
exon 4	1	1.06	0	1	1	0	1	0	0	1	0	0
exon 9	15	15.96	0	15	6	7	0	5	10	4	3	7
exon 11	74	78.72	35	39	42	18	3	29	41	46	7	14
exon 13	9	9.57	3	6	4	5	0	2	7	5	1	1
exon 17	7	7.45	0	7	2	4	0	3	4	0	3	4

**FIGURE 4:**
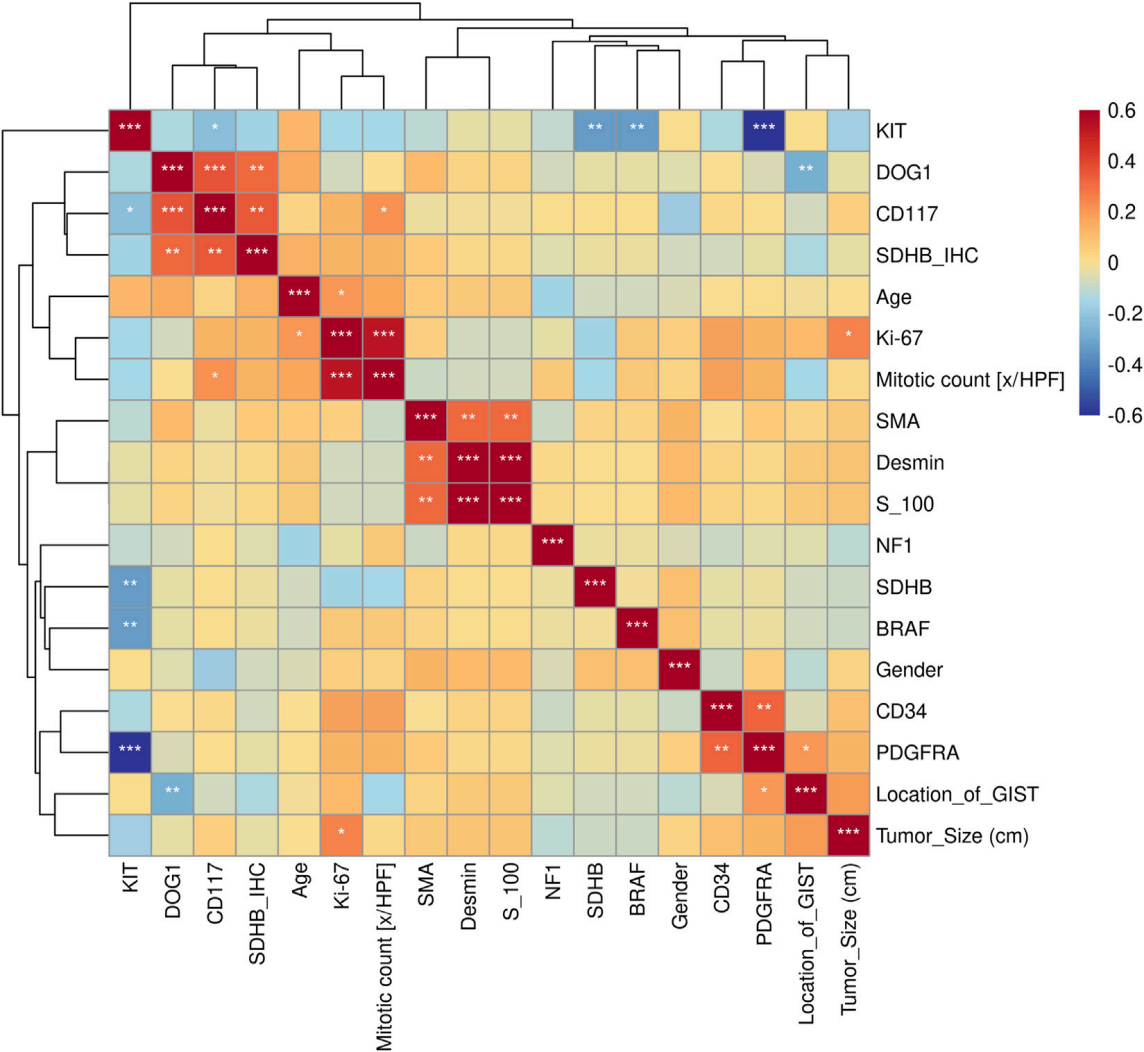
Kendall correlation analysis between gene mutations and clinical features. Test of significance of the Kendall correlation coefficient: “*”, “**”, “***” represent *p* < 0.05, *p* < 0.01, and *p* < 0.001, respectively.

In practice, the diagnosis of GISTs is mainly based on IHC markers, including CD117, CD34, DOG1, and SDHB, and with the help of genetic analysis. Almost all GISTs overexpress CD117 or DOG1 with one exception in our cohort (Supplementary Figure S4). However, the CD117 expression and KIT mutation was not entirely concordant. There was one *KIT*-mutant patient who was CD117-negative but DOG1-positive. DOG1 was universally expressed in three GISTs with *PDGFRA* mutations, while one *PDGFRA*-mutant patient failed to get IHC information. The significance test of the Kendall correlation coefficient showed that the *KIT* mutation was positively correlated with CD117 (*p* < 0.05), while the *PDGFR* mutation was negatively correlated with CD34 (*p* < 0.01) (Figure 4).

**FIGURE 5:**
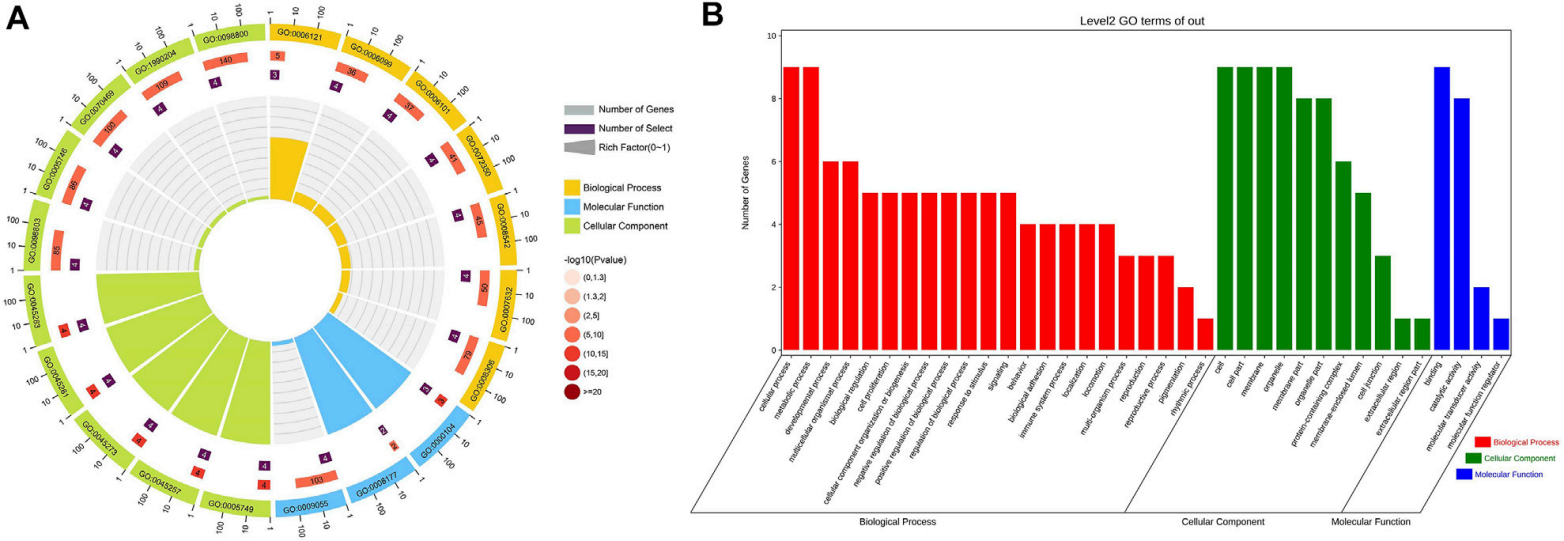
GO functional classification results of nine genes related to GIST **(A)** Circle Diagram of GO Molecular Function Enrichment **(B)** Bar Chart of GO Molecular Function Enrichment.

**FIGURE 6:**
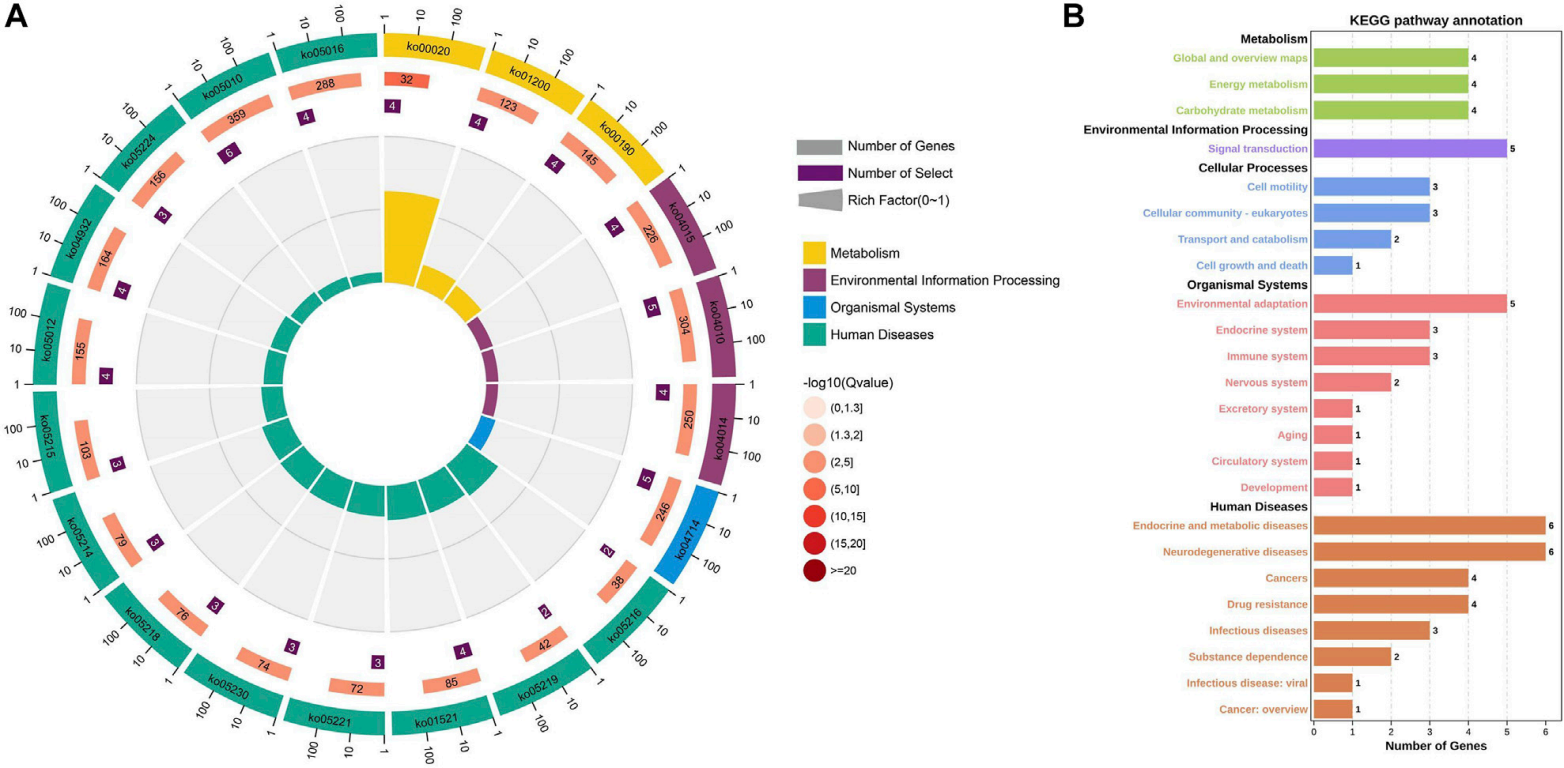
KEGG pathway enrichment analysis of nine genes related to GIST **(A)** Circle Diagram of KEGG pathway enrichment analysis **(B)** Statistics of the enrichment number of the KEGG pathway.

The relationship between clinical features and the immunophenotype was depicted by Figure 4. CD117 (*p* < 0.05) and Ki67 (*p* < 0.001) were positively correlated with patients’ mitotic count. Meanwhile, the age (*p* < 0.05) and tumor size (*p* < 0.01) were positively correlated with Ki67. In addition, the relevance between the tumor location and DOG1 was also positive. GO enrichment analysis was performed on nine GIST-related genes. Biological processes have been enriched by GO analysis. It includes cell proliferation, reproduction, developmental process, and reproductive process, which are related to the growth of cells. This may explain why there are significant correlations among CD117, Ki67, and mitotic count (Figure 5). Meanwhile, KEGG pathway analysis showed that nine GIST-related genes were enriched in cell growth and death, development, aging, environmental adaptation, etc. Perhaps this is why the tumor size is significantly correlated with the Ki67 index (Figure 6).

## Discussion

In the past 10 years, the remedy of GISTs has gradually been evolving from a one-size-fits-all scheme to targeted oncogene treatments for specific molecular GIST subtypes (Blay et al., 2021a). However, the effectiveness of targeted therapy varies among patients because its effectiveness depends on the genetic mutation profile of GIST tumor tissues (Debiec-Rychter et al., 2006). A variety of molecular driven mutations are present in the GIST and are directly related to the curative effect of targeted treatment (von Mehren and Joensuu, 2018b). The most frequent driver mutations occur in *KIT* and *PDGFRA*. In total, 60–70% GISTs can acquire the mutation of *KIT*, and 10–15% GISTs acquire mutation of *PDGFRA* that both promote to the occurrence and development of GISTs. Around 15% of GISTs have other genetic alterations, for example, in SDH family genes, RAS family genes, *BRAF*, *NF1*, or other very rare driver gene mutations (von Mehren and Joensuu, 2018b; Blay et al., 2021b). Therefore, it is necessary to analyze the gene mutation profile of tumor tissues. Although the cost of molecular detection needs to be paid for additionally, the cost of molecular detection is lower than unnecessary adjuvant or neoadjuvant treatment.

In this study, the molecular detection method we chose was targeted next-generation sequencing for nine GIST-related genes. Different from the traditional Sanger sequencing, NGS sequencing can detect molecular variations in multiple genes at the same time. Its outstanding advantage is higher detection sensitivity. NGS sequencing can capture the secondary drug resistance mutation earlier than Sanger sequencing that provides more valuable reference information for the treatment strategies of advanced patients. We found that four patients carried PDGFRA-D842V in 73 primary patients. The aforementioned information suggests that patients should be cautious when choosing IM for treatment. Briefly, in the current study, a gene panel consisting of 158 CDS regions in nine genes, which have clinical interest for the GIST, was tested for targeted sequencing. The genetic profiles of 106 GIST patients were comprehensively analyzed by a 9-gene targeted next-generation sequencing panel. It shows that *KIT* was the most frequent driver gene. Of all patients, 13.21% (14/106) patients had two or more mutations. *KIT* mutations or *PDGFRA* mutations were detected to be mutually exclusive in the GIST. In addition, the significance test of the Kendall correlation coefficient showed that the *KIT* mutation was negatively correlated with the *BRAF* mutation and *SDHB* mutation (*p* < 0.01). Although the analysis results showed a significant correlation, they could not represent the actual clinical situation because there was only one case of *BRAF* and *SDHB* mutation respectively. However, we found similar results in other studies (Corless et al., 2011b; Kondo et al., 2020). Different types of molecular tumor drivers are related to the primary anatomical site. GIST-bearing *KIT* mutations were located in the small intestine (51/94,54.26%) or stomach (36/94,38.30%). All of *KIT* exon 9 mutations were not observed in the stomach. Mutations of *PDGFRA* were observed in the primary gastric GIST.

Our study identified that 88.68% (94/106) of the cases carried *KIT* mutations, 3.77% (4/106) of cases harbored *PDGFRA* mutations, and 7.56% (8/106) of cases were characterized as WT-GISTs. The *KIT* mutation incidence was higher than that reported in a previously published literature study, and the *PDGFRA* mutation incidence was lower than others’ published literature which could be attributed to different sample sizes, populations, and detection sensitivity. The *KIT* mutation was distributed in exons 4, 9, 11, 13, and 17 that contain different types of variation, such as point mutation, duplication, and small fragment deletions. Exon 11 (74/94, 78.72%) accounted for the highest proportion of mutations. The codon mutation (61/94, 64.89%) was the most common variant carried in *KIT* exon 11. In total, 47.54% (29/61) of patients were involved in 557/558 deletion. All patients with the *KIT* exon 9 mutation carried p.Ala502_Tyr503 duplicate mutation except for one missense mutation (p.Ser476Ile) case.

Lincoln SE et al. discovered that the pathogenic germline mutations of tumor susceptibility genes had potential clinical effects, such as clinical trial qualification and earlier detection or prevention of the second primary cancer (Lincoln et al., 2020). We set up a synchronous detection of germline variation to this study and identified different pathogenic germline mutations in two patients from 106 Chinese patients with the GIST. One patient was a wild-type patient without somatic mutation, but one pathogenic germline mutation of *NF1* exon 18 p.R681 * was tested. The other patient detected somatic mutations of *KIT* exon 9 p.A502_Y503dup and exon 17 p.N822Y and developed liver and abdominal metastasis 2 years after initial surgery and IM treatment. The pathogenic mutation KRAS-T58I was detected in the germline molecular assay of this patient. In addition, we also obtained two germline mutations of unknown clinical significance, PDGFRA-S716R and NF1-R2713Q. NF1-L591P was a germline mutation to be identified for the first time and is not recorded in the database.

It is a great pity that there were several limitations to our study. The 9-gene targeted next-generation sequencing panel used in this study was too small for excavation of the complicated genetic alterations in the GIST. Therefore, according to the specific application scenario, there is space for upgrading and optimizing the analysis of the GIST disease occurrence and development and drug efficacy evaluation. Second, in our study, the relapse-free or disease-free survival analysis was not applicable due to the short median follow-up duration. Finally, the functional evaluation of the germline gene variation has not taken a closer study.

In conclusion, our study confirms the utility of the 9-gene targeted next-generation sequencing panel to efficiently identify mutations associated with GISTs. NGS can effectively expand our understanding about the specific mutations of sensitivity in individualized treatment. The occurrence and development of GIST is driven by different molecular variations. Resistance to TKIs arises mainly with resistance mutations in *KIT* or *PDGFRA* which may provide a genetic basis for developing new GIST therapeutic drugs. Our results have important implications for clinical management that supplies reference for clinicians’ individualized diagnosis and treatment.

## Data Availability

The raw data reported in this study has been deposited in the CNGB Nucleotide Sequence Archive (CNSA) (CNSA: https://db.cngb.org/cnsa/) with accession No. CNP0002760.

## Abbreviations

GIST, gastrointestinal stromal tumor; NCCN, National Comprehensive Cancer Network; EUS-FNAB, endoscopic ultrasound with fine-needle aspiration and biopsy; WT, wild-type (no KIT or PDGFRA somatic mutation detected); KIT, mast/stem cell growth factor receptor kit gene; PDGFRA, platelet-derived growth factor receptor A; TKI, tyrosine kinase inhibitor; NGS, next-generation Sequencing; FFPE, formalin-fixed paraffin-embedded.
