# Corrigendum: Molecular Portrait of GIST Associated With Clinicopathological Features: A Retrospective Study With Molecular Analysis by a Custom 9-Gene Targeted Next-Generation Sequencing Panel

**DOI:** 10.3389/fgene.2022.945357

**Published:** 2022-06-28

**Authors:** Haoran Qian, Na Yan, Xiaotong Hu, Junchang Jiang, Zhengzheng Cao, Dan Shen

**Affiliations:** ^1^ Department of General Surgery, Sir Run Run Shaw Hospital, School of Medicine, Zhejiang University, Hangzhou, China; ^2^ Dian Diagnostics Group Co., Ltd., Hangzhou, China; ^3^ Key Laboratory of Digital Technology in Medical Diagnostics of Zhejiang Province, Hangzhou, China; ^4^ Department of Pathology, Sir Run Run Shaw Hospital, School of Medicine, Zhejiang University, Hangzhou, China

**Keywords:** gastrointestinal stromal tumors, molecular subtypes, clinicopathological features, next-generation sequencing, target therapy

In the original article, there was an error in affiliation 3. The corrected affiliation is “Key Laboratory of Digital Technology in Medical Diagnostics of Zhejiang Province, Hangzhou, China.”

In addition, the percentage of *KIT* mutations in GISTs was calculated incorrectly.

A correction has been made to **Abstract**, **Results**, sentence 2. The new sentence reads as follows:

“*KIT* mutations occurred most often in GISTs (94/106, 88.68%), followed by point mutations in *PDGFRA*.”

A correction has been made to **Results**, **Gene Mutation Distributions and Frequencies**, sentence 2. The new sentence reads as follows:

“*KIT* mutations occurred most often in GISTs (94 of 106 tumors, 88.68%), followed by the point mutation in *PDGFRA*.”

The authors apologize for this error and state that this does not change the scientific conclusions of the article in any way. The original article has been updated.

