# Supplementary figures and images for: Molecular Portrait of GISTs Associated With Clinicopathological Features: A Retrospective Study With Molecular Analysis by a Custom 9-Gene Targeted Next-Generation Sequencing Panel
Source: Front Genet. 2022 Apr 25;13:864499. doi: 10.3389/fgene.2022.864499 (PMC9081536; doi:10.3389/fgene.2022.864499)

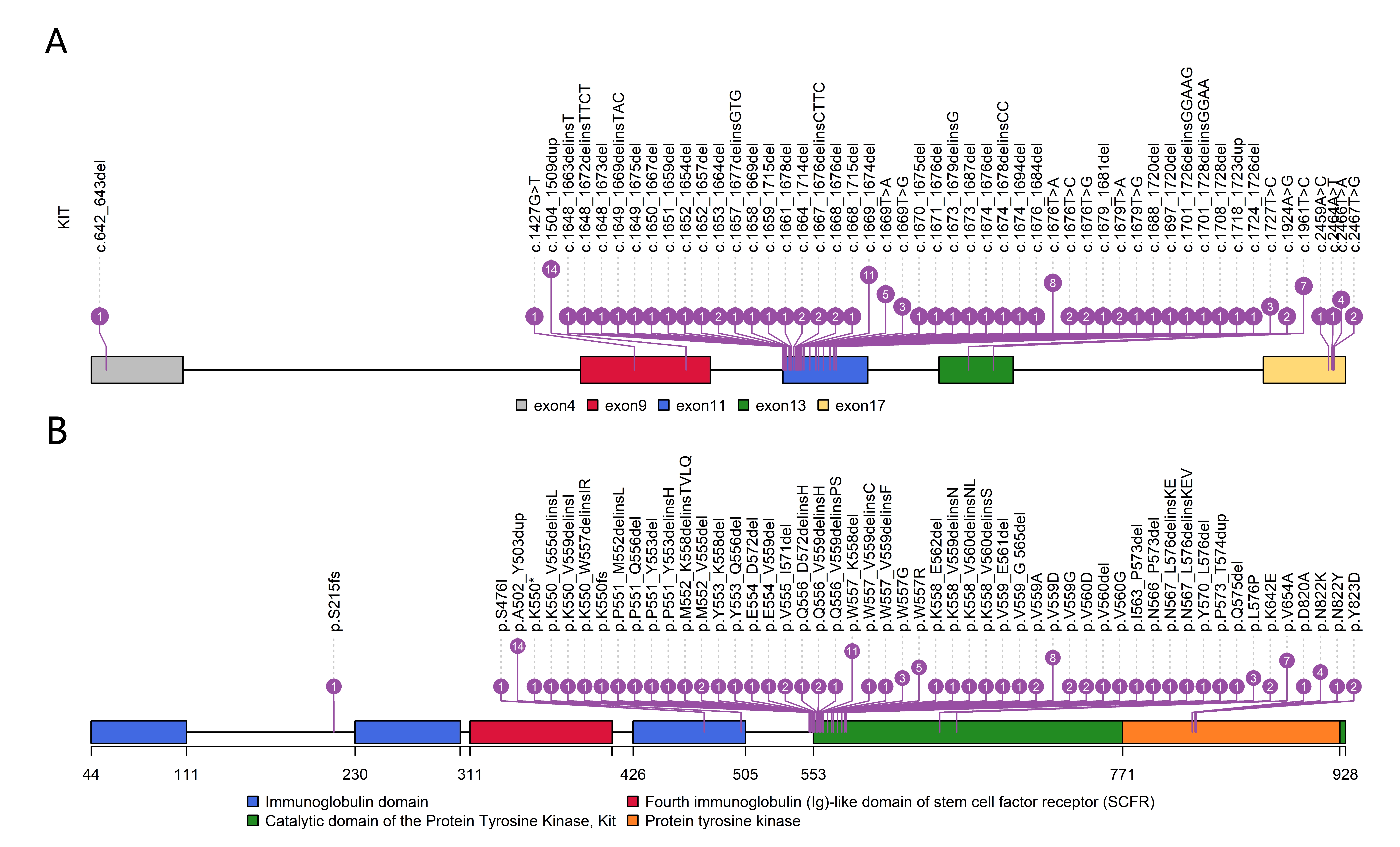

Supplement: Supplementary file 1 [file Image3.JPEG]

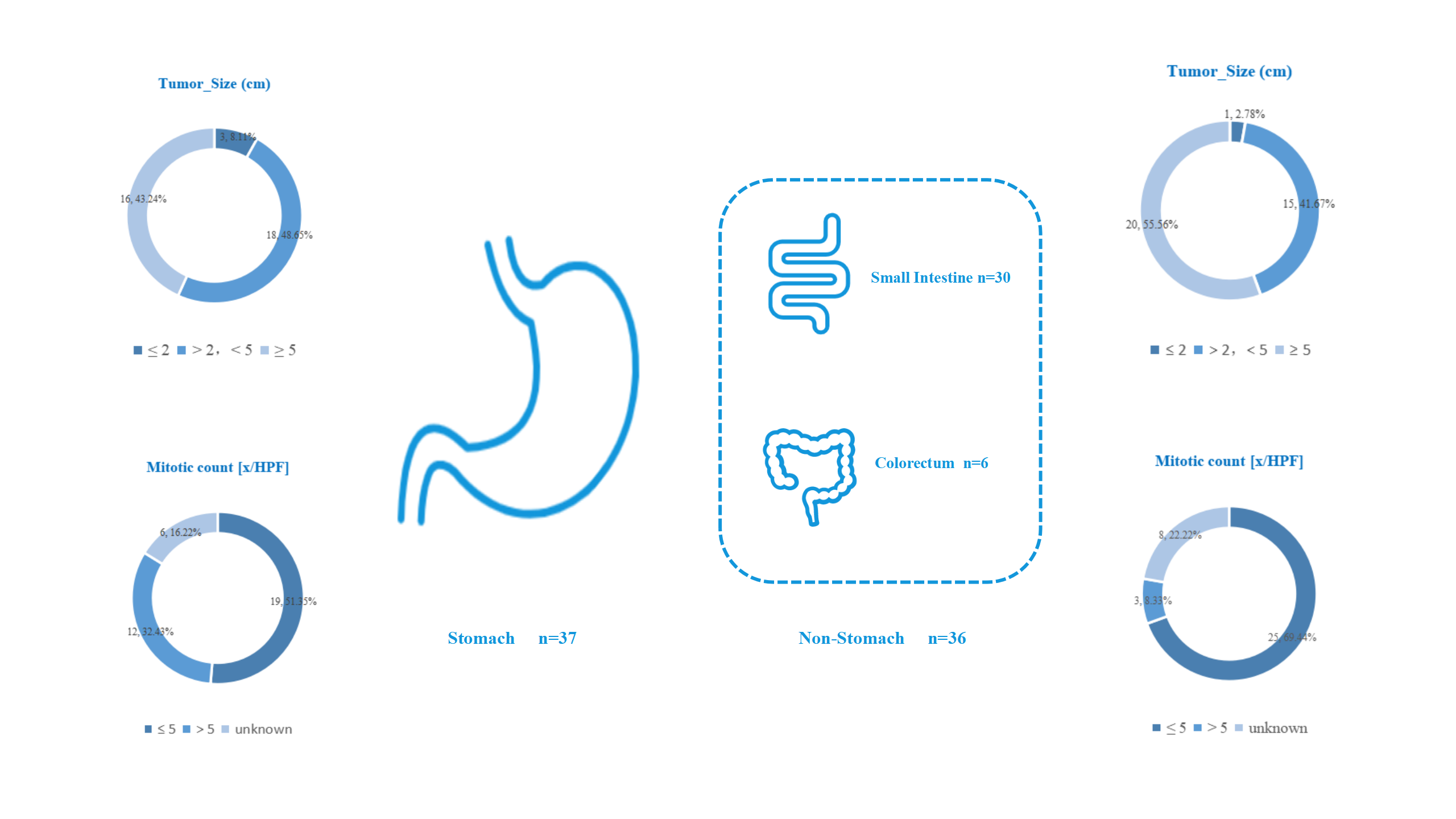

Supplement: Supplementary file 2 [file Image1.TIF]

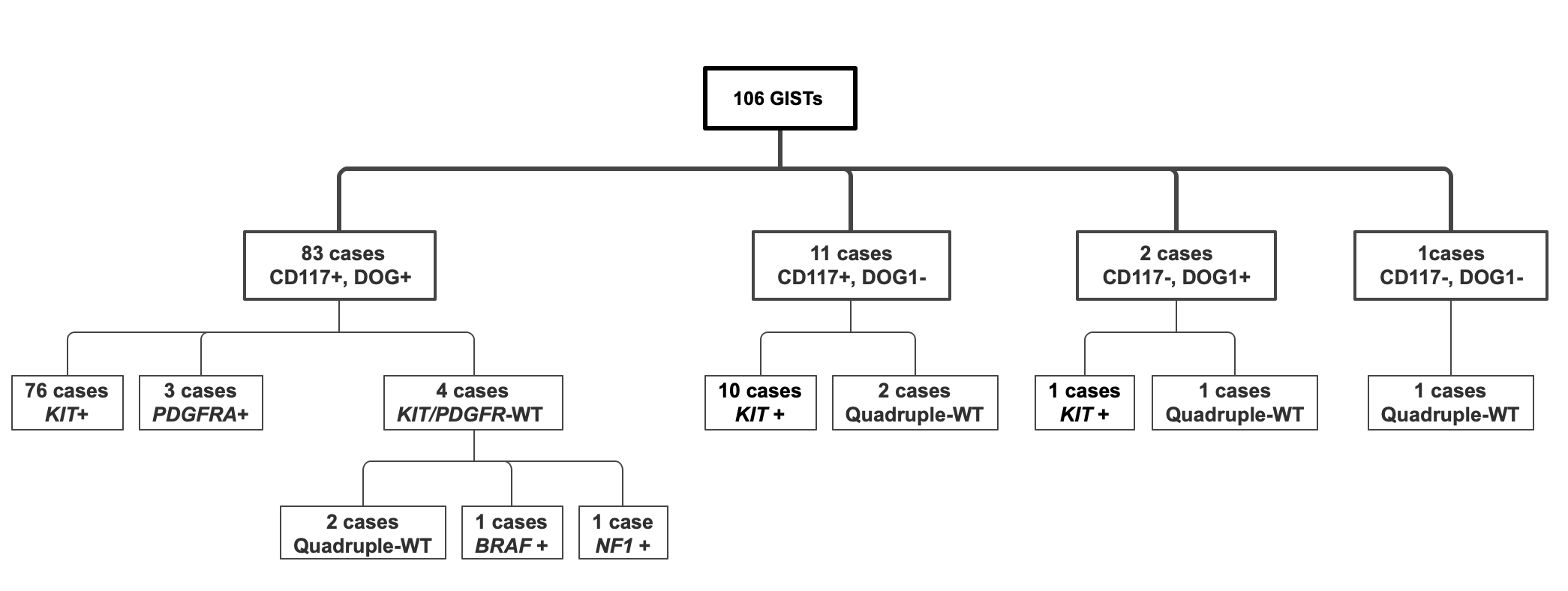

Supplement: Supplementary file 3 [file Image4.PNG]

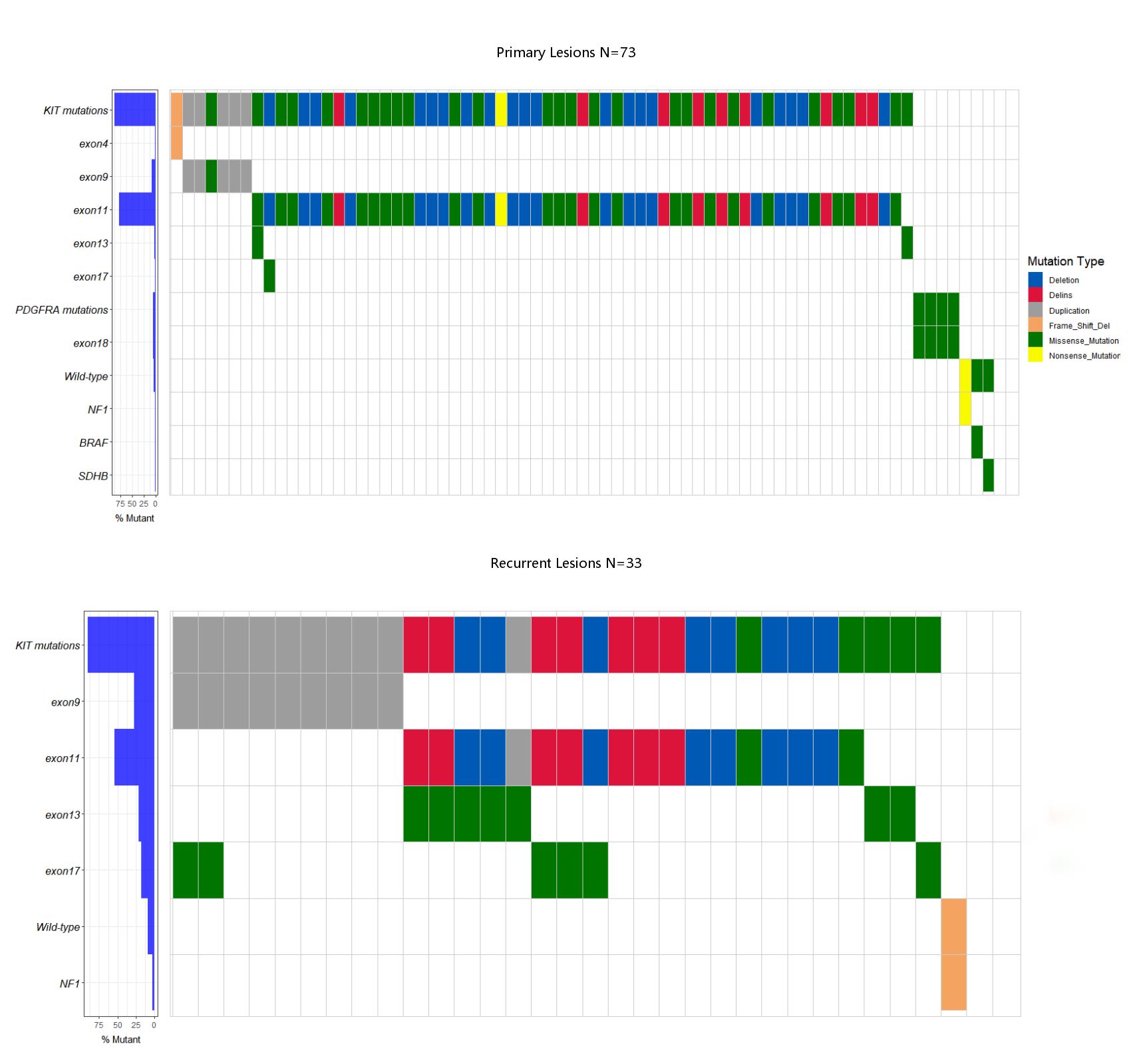

Supplement: Supplementary file 4 [file Image2.PNG]
