# Supplementary material for: Molecular Portrait of GISTs Associated With Clinicopathological Features: A Retrospective Study With Molecular Analysis by a Custom 9-Gene Targeted Next-Generation Sequencing Panel
Source: Front Genet. 2022 Apr 25;13:864499. doi: 10.3389/fgene.2022.864499 (PMC9081536; doi:10.3389/fgene.2022.864499)
Supplement: Supplementary file 5 [file DataSheet2.docx]

Molecular Portrait of GIST associated with clinicopathological features: a retrospective study with molecular analysis by a custom 9-gene targeted next-generation sequencing panel

**SUPPLEMENTARY DATA（Figures can be published online.）**

**Figure S1. Distribution characteristics of tumor size and mitotic count in different location of GIST primary focus.** Location of disease in GIST incidence was not observed as stomach-to-non-Stomach ratio is 1:1.03. The level of mitotic phase in the stomach is higher(Mitotic count [x/HPF] > 5, 32.43%), which is different from non-gastric primary GIST(Mitotic count [x/HPF] > 5, 8.33%；p-value=0.0389). There was no significant difference between the location of primary focus and tumor size.

**Figure S2. The genetic profile of primary** **GIST and resistant GIST.**

**Figure S3.** **Distribution characteristics of KIT somatic mutations.** A: Distribution of mutations in KIT gene. B: Distribution of mutations in KIT functional domains.

**Figure S4. Distribution of immunohistochemical indicators and molecular quotas in 106 GIST patients.**
